# Expression and Genetic Polymorphisms of ERCC1 in Chinese Han Patients with Oral Squamous Cell Carcinoma

**DOI:** 10.1155/2020/1207809

**Published:** 2020-09-26

**Authors:** Chaokui Wang, Ning Gan, Ping Liu, Hongying Chen, Yong Li, Xian Li

**Affiliations:** ^1^The First Affiliated Hospital of Chongqing Medical University, Chongqing Key Lab of Ophthalmology, Chongqing Eye Institute, Chongqing, China; ^2^Stomatological Hospital of Chongqing Medical University, Chongqing, China; ^3^Chongqing Key Laboratory of Oral Diseases and Biomedical Sciences, Chongqing Municipal Key Laboratory of Oral Biomedical Engineering of Higher Education, Chongqing, China

## Abstract

The aim of this study was to investigate the expression of the excision repair cross-complementation group 1 (ERCC1) in oral squamous cell carcinoma (OSCC) and the possible association of ERCC1 polymorphisms with susceptibility and response to chemotherapy of OSCC in a Chinese Han population. The expression of ERCC1 was determined by real-time PCR in eight patients. Four single-nucleotide polymorphisms (SNPs) rs11615, rs3212948, rs3212961, and rs735482 of ERCC1 were genotyped in 113 OSCC patients and 184 healthy controls using a PCR restriction matrix-assisted laser desorption/ionization time of flight mass spectrometry (MALDI-TOF MS) assay. We found that a higher gene expression of ERCC1 was observed in tumor tissue as compared to pericarcinomatous tissue in OSCC patients. All genotypic and allelic frequencies of the tested ERCC1 polymorphisms were in Hardy-Weinberg equilibrium. The genotypic and allelic frequencies of rs11615, rs3212948, rs3212961, and rs735482 of ERCC1 were not different between OSCC patients and controls. No correlation was observed between ERCC1 polymorphisms and the response to chemotherapy. Our results show that ERCC1 is increased in the tumor tissue of OSCC patients. The investigated ERCC1 gene polymorphisms (rs11615, rs3212948, rs3212961, and rs735482) are not associated with the susceptibility and response to chemotherapy of OSCC in our investigated Chinese Han population.

## 1. Introduction

Head and neck squamous cell carcinoma (HNSCC) is the sixth most common cancer in the world. Oral squamous cell carcinoma (OSCC) is the most common type of HNSCC, and China has one of the highest incidences of this type of cancer [1]. Although the exact etiology of the disease remains unclear, several studies revealed that environmental risk factors and genetic factors play a significant role in the onset and development of OSCC [2].

Recent studies have suggested that genetic factors are associated with the risk of OSCC, including polymorphisms of CYP1A1, EC-SOD, GSTT1, GSTM1, and ALDH2 [3, 4]. Environmental factors such as smoking and drinking history are also associated with the susceptibility to OSCC [4]. Gelsolin rs1078305 and rs10818524 polymorphisms were also reported to be associated with the risk of OSCC in a Chinese Han population [5]. Furthermore, a joint effect of WISP1 rs2929970 with smoking as well as WISP1 rs16893344 with betel nut chewing was reported to contribute to the occurrence of OSCC [6]. The exact role of these genetic factors in the pathogenesis of OSCC is not well understood, and more studies are needed using larger sample sizes in different ethnic populations.

DNA repair is critical for maintaining DNA stability and integrity and cell function [7, 8]. To date, four major DNA repair pathways have been identified: base excision repair (BER), nucleotide excision repair (NER), double-strand break repair (DSBR), and mismatch repair (MMR) [8, 9]. Excision repair cross-complementation group 1 (ERCC1) is the key component in the nucleotide excision repair (NER) pathway and plays a crucial role in the process of excision repair. It has been reported that ERCC1 gene polymorphisms are associated with the risk of some cancers, such as pancreatic cancer [10], nasopharyngeal carcinoma [11], lung cancer [12], melanoma [13], childhood gastric cancer [14], and childhood acute lymphoblastic leukemia [15]. Gao et al. reported that ERCC1 500C>T alone (including rs11615 and rs3212948) was more frequent in Caucasians with melanoma than in healthy Caucasians [13]. Zhu et al. reported that ERCC1 rs3212948 is a lung cancer risk-associated polymorphism through a systematic review and meta-analysis [12]. The variant allele of rs3212961 was shown to be associated with risk of lung cancer among long-term smokers [16], and the ERCC1 rs735482 A allele was associated with the tumor size of hepatocellular carcinoma [17].

Platinum-based therapy is commonly used in the treatment of several human cancers by damaging DNA in cancer cells at interphase. It has been reported that the ERCC1 C19007T (rs11615) polymorphism is associated with the response to platinum-based chemotherapy in several types of cancers [18, 19]. The ERCC1 rs11615 genotype was found to be significantly associated with treatment response to chemotherapy in patients with advanced non-small-cell lung cancer [20]. A Chinese study reported that a polymorphism of ERCC1 could influence the response to platinum-based chemotherapy, whereby the ERCC1 C/C genotype is a favorable factor for the platinum-based chemotherapy response in Chinese patients with non-small-cell lung cancer [21]. In a meta-analysis, it was concluded that ERCC1 C118T (rs11615) and C8092A could predict both the objective response rate as well as overall survival for platinum-based chemotherapy in Asian NSCLC patients through the analysis of 33 studies including 5373 patients [22]. One study from Taiwan found that the ERCC1 rs735482 CC genotype is associated with an increased risk of recurrence in male patients with OSCC treated with concurrent chemoradiotherapy [23].

Until now, no studies have been reported concerning the association of ERCC1 gene polymorphisms and OSCC, and only one has addressed the role of ERCC1 rs735482 in the response to chemoradiotherapy in OSCC [23], and this was therefore the subject of the study reported here. In view of earlier studies on ERCC1 gene associations with cancer as well as the response to chemotherapy, we chose four ERCC1 SNPs including rs11615, rs3212948, rs3212961, and rs735482 in our study.

## 2. Materials and Methods

### 2.1. Study Subjects

The study subjects consisted of 113 OSCC patients and 184 healthy normal controls. The diagnosis of OSCC was histologically confirmed by the pathology department. Patients and controls were recruited from the Oral and Maxillofacial Surgery Department, Stomatological Hospital of Chongqing Medical University, between June 2013 and November 2014. The patients were followed up until June 2018, with an average follow-up time of 34 months (range from 24 months to 60 months). All patients were followed up by telephone or during their visit to our outpatient clinic every three-six months until death or the end of the study. To study the gene expression of ERCC1 in the tumor tissue of OSCC patients, tumor and corresponding adjacent normal tissues (pericarcinomatous) were collected from eight randomly selected patients. Written informed consents were obtained from each subject. All procedures followed the tenets of the Helsinki declaration and were approved by the Clinical Ethical Research Committee of the Stomatological Hospital of Chongqing Medical University. The demographic and clinical features of the patients and controls are presented in Table 1. In brief, there was no significant difference regarding age and smoking status between OSCC patients and controls. However, there was a significant male preponderance and drinking prevalence in OSCC patients compared to normal controls.

### 2.2. Chemotherapy Regimen and Response Assessment

Patients were treated with the following cisplatin-based combination chemotherapy regimen: cisplatin+5-fluorouracil+docetaxel. This chemotherapy treatment was repeated every three weeks. The treatment was continued for a maximum of five cycles. The treatments were suspended in case of disease progression or unacceptable toxicity. The treatment outcome was evaluated as based on the published response evaluation criteria in solid tumors [24]. Complete remission (CR) and partial remission (PR) to chemotherapy were considered as good response, and stable disease (SD) and progressive disease (PD) to chemotherapy were regarded as nonresponse.

### 2.3. Genomic DNA Preparation and Genotyping

Genomic DNA was isolated from blood leukocytes using the Tiangen DNA blood genome extraction kit (Tiangen, Beijing, China). The extracted DNA was stored at -80°C until use. Gene polymorphisms were genotyped using the matrix-assisted laser desorption/ionization time of flight mass spectrometry (MALDI-TOF-MS) by the Sangon Biotechnology Company (Shanghai, China).

### 2.4. Real-Time PCR

Total RNA was extracted from tumor and pericarcinomatous tissues using Takara RNAios Plus (Takara, Dalian, China) according to the manufacturer's instructions. Reverse transcription of RNA was performed using the Superscript III Reverse Transcriptase system (Takara, Dalian, China). Real-time quantitative PCR was performed on the iCycler (Bio-Rad, Veenendaal, The Netherlands) using the Quanti Tect SYBR Green PCR kit (Applied Biosystems). The forward and reverse primer sequences used were beta-actin forward, 5′-GGATGCAGAAGGAGATCACTG-3′, reverse, 5′-CGATCCACACGGAGTACTTG-3′. ERCC1 forward 5′-GGGAATTTGGCGACGTAATTC-3′, reverse 5′-GCGGAGGCTGAGGAACAG-3′. The relative expression level of ERCC1 was calculated using the 2^−ΔΔCt^ method.

### 2.5. Statistical Analysis

Deviations from the Hardy-Weinberg equilibrium (HWE) were evaluated by the chi-square test. Differences of the ERCC1 gene allele and genotype frequencies between groups were analyzed using the chi-square test. The paired-sample *T* test was used to analyze the ERCC1 gene expression between tumor and pericarcinomatous tissues. All data were analyzed using SPSS17.0. The Bonferroni correction was used for SNP analysis. A *P*_c_ < 0.05 was considered statistically significant.

## 3. Results

### 3.1. Expression of ERCC1 in Carcinoma Tissue of OSCC Patients

**Figure 1:**
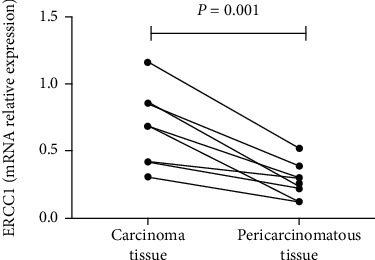
ERCC1 mRNA level in the carcinoma tissue and corresponding pericarcinomatous tissue of OSCC patients (*n* = 8) was measured by real-time PCR. Data were normalized to *β*-actin and are presented as mean ± S.E.M. The paired-sample *T* test was used for statistical analysis.

In this study, we examined the gene expression of ERCC1 in the carcinoma tissue and corresponding normal pericarcinomatous tissue of OSCC patients using real-time PCR. The results showed that the gene expression of ERCC1 in carcinoma tissue (0.67 ± 0.29) was significantly higher than that in corresponding normal pericarcinomatous tissue (0.27 ± 0.13, *P* = 0.001) of OSCC patients (Figure 1).

### 3.2. Genetic Polymorphisms of ERCC1 in OSCC Patients

We investigated the associations of four different ERCC1 SNPs with the risk of developing OSCC. The four SNPs of ERCC1were successfully genotyped in all the patients and controls. All genotype and allele frequency distributions were in Hardy-Weinberg equilibrium (*P* > 0.05). The distribution of allele and genotype frequencies of the four tested ERCC1 polymorphisms showed that the genotypic and allelic frequencies of rs11615, rs3212948, rs3212961, and rs735482 of ERCC1 were not different between OSCC patients and normal controls (Table 2). These SNPs were also stratified according to gender and drinking, but no statistically significant associations could be detected (data not shown).

As mentioned above, ERCC1 polymorphisms have been shown to be associated with the response to chemotherapy in certain cancers. We therefore also analyzed the association of ERCC1 polymorphisms with the response to chemotherapy. We were not able to retrieve 30 of our OSCC patients, and analysis was therefore confined to the remaining 83 patients. At the end of the follow-up, 47 OSCC patients showed a good response to cisplatin-based chemotherapy, with a response rate of 56.6%. We did not find a significant association between rs11615, rs3212948, rs3212961, or rs735482 ERCC1polymorphisms and the response to chemotherapy in OSCC (Table 3).

## 4. Discussion

In this study, we showed that the ERCC1 mRNA level was significantly increased in OSCC carcinoma tissue as compared to the corresponding normal pericarcinomatous tissue. We failed to detect an association between four SNPs of ERCC1 with susceptibility to OSCC or the response to chemotherapy.

A study in hepatocellular carcinoma (HCC) patients showed that the level of ERCC1 in the cancer tissues was significantly lower than that in adjacent paracancer tissues and that the expression of ERCC1 was negatively associated with hepatic capsular and microvascular invasion [25]. The difference concerning the expression of ERCC1 in several cancers may be due to the different types of cancer and/or the different roles of ERCC1 in these various tumors. It has been reported that a high ERCC1 expression may predict cisplatin-based chemotherapy resistance and poor outcome in unresectable squamous cell carcinoma of the head and neck [26]. These data collectively suggest that an increased ERCC1 expression may result in more resistance to cisplatin-based chemotherapy and poor outcome. In our study, the OSCC carcinomas were all completely resected as shown by pathological examination. In a future study, we plan to do a more comprehensive analysis on the association between the expression of ERCC1 expression with overall survival and relapse-free survival times in a larger patient population.

Although the etiology and pathogenesis of OSCC are not fully understood, genetic polymorphisms are increasingly recognized as important risk factors for the development of these carcinomas. Recent studies have suggested that some SNPs of the ERCC1gene may be a risk factor for cancer aggressiveness [10, 27, 28], although we could not confirm this for OSCC. As there are many SNPs in the ERCC1 gene and only a few SNPs may contribute to the occurrence of cancer, it is extremely important to choose the right candidate polymorphisms to test an association with OSCC. In view of the previous reports, we chose four SNPs in the ERCC1 gene including rs11615, rs3212948, rs3212961, and rs735482 which are reported to be associated with certain cancers [12, 13, 20, 23]. In our study, we did not found a significant association between four SNPs in the ERCC1 gene and susceptibility of OSCC.

Emerging evidence suggests polymorphisms in ERCC1 may help predict response to cisplatin and other platinum-based chemotherapeutics. Multiple ERCC1 single-nucleotide polymorphisms (SNPs) have been associated with platinum chemotherapy response [19, 29, 30]. A study investigating the role of three SNPs of ERCC1 in the clinical outcome of non-small-cell lung cancer (NSCLC) showed that the TT genotype of ERCC1 rs11615 and the AA genotype of rs3212986 polymorphisms were associated with an increased risk of death from NSCLC [31]. We were not able to show a significant association between rs11615, rs3212948, rs3212961, and rs735482 ERCC1 polymorphisms and response to chemotherapy in OSCC. The discrepancy is not clear and as mentioned earlier may be due to ethnicity or type of cancer.

There are a number of weak points in our study. First, the sample size is relatively small, so that it may not have had sufficient power to find a statistically significant association of the tested SNPs with susceptibility and response to chemotherapy in OSCC. Despite the small number of samples, we found a significant statistical difference concerning the ERCC1 mRNA level in the carcinoma tissue as compared to the corresponding pericarcinomatous tissue of OSCC patients, but further investigation with a larger sample size needs to be performed to confirm our results. Second, we did not cover the complete variation of the ERCC1 gene sequence, which means that our study does not exclude the possibility that other SNPs within the ERCC1 gene might be associated with OSCC. Third, the patients and controls were all Chinese Han, which means that there is a possibility that the tested SNPs may show an association with other ethnic populations. More studies are needed to clarify these issues.

In conclusion, our study found that the expression of ERCC1 was increased in OSCC carcinoma tissue. However, we were not able to detect an association between ERCC1 gene polymorphisms and OSCC in a Chinese Han population. We also did not find an association between ERCC1gene polymorphisms and the response to chemotherapy in OSCC. Discrepancies with earlier reports may be due to ethnic differences, and further studies with larger sample sizes in different ethnic populations are needed to study the exact role of ERCC1 polymorphisms in OSCC carcinogenesis.

## Figures and Tables

**Table 1:** Clinical characteristics of OSCC patients and controls.

Characteristics	OSCC patients (*n* = 113)	Normal controls (*n* = 184)	*P* value
Age (year)			
<60	49 (43.4)	100 (54.3)	0.074
≥60	64 (56.6)	84 (45.7)	
Gender (*n*, %)			
Male	78 (69.0)	96 (53.2)	0.005
Female	35 (31.0)	88 (46.8)	
Smoking status			
Never	52 (46.0)	98 (53.3)	0.234
Ever	61 (54.0)	86 (46.7)	
Drinking status			
Never	75 (66.4)	148 (80.4)	0.009
Ever	38 (33.6)	36 (19.6)	
TNM stage			
I/II	53		
III/IV	60		
Lymph node metastasis			
Negative	109		
Positive	4		

**Table 2:** Frequencies of genotypes and alleles of ERCC1 polymorphisms in OSCC patients and controls.

SNP	Genotype allele	OSCC (*N* = 113)	Controls (*N* = 184)	*P* value	*P*_c_	OR	95% CI	
rs11615	CC	60	111	0.221	NS	0.745	0.464	1.195
	CT	48	66	0.256	NS	1.320	0.817	2.133
	TT	5	7	0.792	NS	1.171	0.362	3.781
	C	168	288	0.271	NS	0.805	0.546	1.186
	T	58	80	0.271	NS	1.243	0.843	1.832

rs3212948	CC	5	7	0.792	NS	1.171	0.362	3.781
	CG	48	66	0.256	NS	1.320	0.817	2.133
	GG	60	111	0.221	NS	0.745	0.464	1.195
	C	58	80	0.271	NS	1.243	0.843	1.832
	G	168	288	0.271	NS	0.805	0.546	1.186

rs3212961	AA	22	42	0.495	NS	0.817	0.458	1.459
	AC	60	91	0.542	NS	1.157	0.724	1.849
	CC	31	51	0.958	NS	0.986	0.584	1.666
	A	104	175	0.716	NS	0.940	0.674	1.310
	C	122	193	0.716	NS	1.064	0.763	1.483

rs735482	AA	34	57	0.872	NS	0.959	0.576	1.596
	AC	58	83	0.297	NS	1.283	0.802	2.052
	CC	21	44	0.281	NS	0.726	0.406	1.301
	A	126	197	0.598	NS	1.094	0.784	1.526
	C	100	171	0.598	NS	0.914	0.655	1.276

CI: confidence intervals; OR: odds ratios; NS: not significant; *P*_c_: the Bonferroni correction *P* values.

**Table 3:** Association between ERCC1 polymorphisms and response to chemotherapy in OSCC patients.

SNP	Total frequencies	CR+PR	SD+PD	*P* value	OR (95% CI)
rs11615					
CC	45	24	21	Ref	—
CT	35	22	13	0.495	1.481 (0.601-3.648)
TT	3	1	2	0.601	0.438 (0.037-5.177)
rs3212948					
GG	45	24	21	Ref	—
CG	35	22	13	0.495	1.481 (0.601-3.648)
CC	3	1	2	0.601	0.438 (0.037-5.177)
rs3212961					
CC	22	12	10	Ref	—
AC	45	27	18	0.793	1.25 (0.446-3.500)
AA	16	8	8	1	0.833 (0.229-3.028)
rs735482					
AA	24	14	10	Ref	—
AC	44	27	17	1	1.134 (0.412-3.125)
CC	15	6	9	0.333	0.476 (0.128-1.771)

## Data Availability

The data used to support the findings of this study are included within the article.
